# Use of a Text Message Program to Raise Type 2 Diabetes Risk Awareness and Promote Health Behavior Change (Part I): Assessment of Participant Reach and Adoption

**DOI:** 10.2196/jmir.2928

**Published:** 2013-12-19

**Authors:** Lorraine R Buis, Lindsey Hirzel, Scott A Turske, Terrisca R Des Jardins, Hossein Yarandi, Patricia Bondurant

**Affiliations:** ^1^University of MichiganDepartment of Family MedicineAnn Arbor, MIUnited States; ^2^Wayne State UniversityCollege of NursingDetroit, MIUnited States; ^3^Southeast Michigan Beacon CommunityDetroit, MIUnited States; ^4^Greater Cincinnati Beacon CollaborativeCincinnati, OHUnited States

**Keywords:** diabetes mellitus, type 2, mobile health, cellular phone, text messaging, risk reduction behavior, program evaluation

## Abstract

**Background:**

There are an estimated 25.8 million American children and adults, equivalent to 8.3% of the US population, living with diabetes. Diabetes is particularly burdensome on minority populations. The use of mobile technologies for reaching broad populations is a promising approach, given its wide footprint and ability to deliver inexpensive personalized messages, to increase awareness of type 2 diabetes and promote behavior changes targeting risk factors associated with type 2 diabetes. As a part of the Beacon Community Cooperative Agreement Program, txt4health, a public-facing mobile health information service, was launched in 3 Beacon Communities: the Southeast Michigan Beacon Community in Detroit, MI, the Greater Cincinnati Beacon Community in Cincinnati, OH, and the Crescent City Beacon Community in New Orleans, LA. Txt4health is a mobile health information service designed to help people understand their risk for type 2 diabetes and become more informed about the steps they can take to lead healthy lives.

**Objective:**

The purpose of this investigation was to use the RE-AIM framework to document txt4health reach and adoption by focusing on enrollment and participant engagement in program pilots in Southeast Michigan and Greater Cincinnati.

**Methods:**

We conducted a retrospective records analysis of individual-level txt4health system data from participants in Southeast Michigan and Greater Cincinnati to determine participant usage of txt4health and engagement with the program.

**Results:**

Results from the retrospective records analysis revealed that 5570 participants initiated the 2-step enrollment process via 1 of 3 enrollment strategies: text message, website, or directly with Beacon staff who signed participants up via the website. In total, 33.00% (1838/5570) of participants completed the 2-step enrollment process and were fully enrolled in the program. All participants (100.00%, 1620/1620) who enrolled via text message completed the entire 2-step enrollment process versus 5.52% (218/3950) of participants who enrolled via website or a Beacon staff member. Of those who fully enrolled, 71.00% (1305/1838) completed the diabetes risk assessment and 74.27% (1365/1838) set an initial weight loss goal. Overall, 39.06% (718/1838) of participants completed all 14 weeks of the program and 56.26% (1034/1838) dropped out before completing all 14 weeks, with the bulk of dropouts occurring in the first 4 weeks. Length of participation varied greatly, ranging from 0-48.7 weeks (median 8.6, mean 15.8, SD 15.8). Wide variability of participant engagement in regards to weekly weight and physical activity was documented.

**Conclusions:**

Although broadly focused public health text message interventions may have the potential to reach large populations and show high levels of engagement among some users, the level of individual engagement among participants varies widely, suggesting that this type of approach may not be appropriate for all.

## Introduction

According to 2011 estimates from the Centers for Disease Control and Prevention, there are 25.8 million American children and adults, the equivalent of 8.3% of the US population, living with diabetes [1]. Moreover, 79 million American adults older than 20 years of age (35%) have prediabetes, a condition characterized by higher than normal blood glucose or glycated hemoglobin levels (a measure of long-term blood sugar control) not yet in the diabetic range [1]. Prediabetes is associated with increased risk for developing type 2 diabetes, heart disease, and stroke [1]. These staggering rates pose a population-level health problem because diabetes is a leading cause of heart disease and stroke, and it is the seventh leading cause of death in the United States [1]. Diabetes is particularly burdensome on minority populations, including African Americans [2-4]; after adjusting for population age differences, 12.6% of non-Hispanic African American adults older than 20 years have diagnosed diabetes and a 77% greater risk of diabetes diagnosis compared to non-Hispanic White adults [1].

Targeting diabetes prevention and self-management of current diabetes is essential for reducing health care expenditures. The total costs attributed to diabetes in the United States is estimated at $174 billion and estimated medical expenses for diabetics are more than double that of patients without diabetes [1]. Results from the Diabetes Prevention Program (DPP) indicate that counseling and behavior changes that result in modest weight loss and increased physical activity dramatically lower the risk of developing type 2 diabetes, and are more effective than the pharmacologic intervention metformin in the short term and comparable in the long term [5,6]. Despite the effectiveness of the DPP, costs to deliver the program are high. As a result, numerous attempts to adapt and translate the program into alternative, more affordable settings have been made [7-9] and these approaches have been shown to be effective at promoting similar results [10-12]. One strategy for promoting behavior change with the potential to reach a large population is through the use of mobile health (mHealth) interventions delivered via cell phone. With the ubiquity of cell phones in the United States, mHealth approaches have been gaining momentum as a viable intervention delivery modality.

According to recent estimates from the Pew Internet & American Life Project, 91% of American adults own a cell phone and 56% own a smartphone [13]. Moreover, among cell phone users, 80% have used their phone for text messaging, 43% have downloaded apps, and 31% have looked for health or medical information online [14]. The use of mobile approaches for reaching broad populations is a promising strategy given the high penetration and interactive capability of cell phones across diverse populations, particularly in African American and Latino populations, which have traditionally experienced great health disparities. Cell phone adoption has become so pervasive that access in low-income groups is also common, with 86% of adults with annual household incomes below $30,000 owning a cell phone [15]. Evidence for the use of mHealth to support behavior change is growing. Systematic reviews of cell phone behavior change interventions utilizing text messaging have shown positive behavior change [16,17], and text message interventions for diabetes [18-22], smoking cessation [23-26], medication adherence [19,21,27,28], and weight loss [29-31] have been documented.

To raise awareness of type 2 diabetes at the population level and to inform individuals of their risk for developing type 2 diabetes, 3 pilots of txt4health, a free automated and personalized 14-week text message program focused on diabetes, were launched in the Detroit, MI, Cincinnati, OH, and New Orleans, LA metropolitan areas through funding from the Beacon Community Cooperative Agreement Program [32]. The purpose of this 2-part investigation was to evaluate the txt4health pilots in Southeast Michigan and Greater Cincinnati through the lens of the RE-AIM framework. In the present paper (Part I), we seek to document the program’s reach and adoption; in Part II, we seek to document the program’s efficacy in terms of perceptions of program satisfaction, ease of use, and usefulness. In comparison to the majority of previous work that has focused on small scale implementations of mHealth programs [33,34], this txt4health evaluation represents an effort to understand user usage and perceptions of a program operating at scale.

##  Methods

### Overview

This evaluation of txt4health was conducted in 2 parts. In Part I, we conducted a retrospective records analysis of individual-level txt4health system usage data from participants in Southeast Michigan and Greater Cincinnati to determine participant usage of the program, with specific focus on intervention reach and participant adoption. This is the focus of the present paper. In Part II, we conducted a multimodal user survey with Southeast Michigan and Greater Cincinnati txt4health users recruited through txt4health to understand participant perceptions of program satisfaction, use, and behavior change as a result of using txt4health. Results from Part II of this evaluation are provided in the companion paper.

### Program Description

Txt4health is an automated, personalized, interactive text message service with a primary goal of helping people understand their risk for type 2 diabetes by offering a diabetes risk assessment. Txt4health further seeks to inform users about the steps they can take to reduce their diabetes risk through sending individually tailored messages over 14 weeks. These messages are tailored according to an individual’s diabetes risk profile and focus on diet and exercise, connections to local resources, and educational messages that promote behavior change through weight and physical activity self-monitoring. Moreover, txt4health participants are encouraged to participate in weekly weight and physical activity tracking. Although the cost to register for txt4health was free to participants, standard text message rates applied. The txt4health intervention has been more fully described previously [32]. To be eligible to participate in the txt4health pilots, users had to self-report residing in a ZIP code in 1 of the 7 Southeast Michigan Beacon Community (SEMBC) or the 16 Greater Cincinnati Beacon Collaborative (GCBC) counties.

### Program Enrollment

To enroll in txt4health, users were required to complete a 2-step enrollment process, which could be completed in 1 of 3 ways. First, participants could initiate enrollment through texting the word “health” to the short code 300400, which generated an automated text response requiring users to text back their ZIP code to validate their participation and complete the enrollment process. Second, participants could enter their cell phone number and ZIP code into an online enrollment utility available on the txt4health website. Once registered online, an automated text response requesting users to validate their cell phones by texting a reply was generated and sent via text message to the participant’s cell phone. Third, participants could initiate enrollment through providing a cell phone number and ZIP code directly to SEMBC and GCBC staff members who entered this information for potential participants into the online enrollment utility. This would then generate the same automated text response requiring users to validate their cell phone and confirm their participation by texting a response to txt4health. Enrollment procedures have been fully described previously [32].

Upon enrollment in txt4health, participants were offered a diabetes risk assessment that included an optional health profile, consisting of self-reported weight and height, from which a suggested weight loss goal was calculated (for individuals for whom weight loss was recommended). Participants were then asked to set a target weight goal. Once the health profile was complete, participants were asked to complete an optional 8-item diabetes risk assessment via text message (1 question per text message). Diabetes risk assessment items included self-reported age, daily physical activity level, gender, gestational diabetes screen of having diabetes while pregnant or having given birth to a baby more than 9 lb (if the participant was a woman), sibling history of diabetes, parental history of diabetes, ethnicity, and smoking status. See Table 1 for the full list of assessment items.

Although participants were asked to complete the health profile and diabetes risk assessment questions, these were not required for enrollment. Once enrolled, participants received approximately 5 to 7 messages per week for 14 weeks. Depending on an individual’s health profile and the level of interactivity, participants received tailored messages from 1 of 2 message streams: high risk messages or low/unknown risk messages. At the end of each week, all participants were asked to report their current weight and the number of days they were physically active over the past week. Individuals also received geographically localized text messages that included information on issues and events locally relevant to the audience (ie, advertisement of local health fairs, health-related resources, or time-sensitive subject matters). Finally, every 4 weeks, participants received a text message that reiterated instructions on how to opt out of txt4health or to access assistance.

**Table 1:** Items from the txt4health diabetes risk assessment.

Topic	Message from txt4health
Age	This is a sensitive question, but stick with me, it’s important. How old are you? Reply with your current age in years (for example, 57).
Physical activity	How much exercise do you get in a usual day? Reply 1 if you get little or no exercise or Reply 2 if you are very active most days.
Gender	In order for me to give you helpful information, I need to know if you are a male or a female. Reply 1 for Male or 2 for Female.
Gestational diabetes screen	Your past helps determine your risk for diabetes. Did you have diabetes while pregnant OR give birth to a baby over 9 lbs? Reply YES or NO.
Family history (sibling)	Tell me about your family history. Do you have a brother or sister with diabetes? Reply 1 for Yes; 2 for No; or 3 if you do not know.
Family history (parental)	You only have 3 more questions left! What about your parents? Do either of them have diabetes? Reply 1 for Yes; 2 for No; or 3 if you do not know.
Ethnicity	What is your ethnicity? Reply 1 for White; 2 for Black/African American; 3 for Hispanic/Latino; 4 for Asian/Pacific Islander; 5 for Other.
Smoking status	Let’s talk about smoking for a minute. You can be honest with me. Do you smoke cigarettes? Reply YES or NO.

###  Program Rollout

The txt4health pilots launched in February 2012. In Southeast Michigan, txt4health was launched as a part of a larger, broad-based public health campaign called Fighting D in the D, a public health campaign launched by the SEMBC in the greater Detroit area to promote type 2 diabetes awareness. Txt4health was used as a call to action within the larger Fighting D in the D campaign. This campaign began with a kickoff event featuring a keynote address by US Surgeon General Regina Benjamin, MD, and a community roundtable featuring health care leaders of Southeast Michigan. The campaign was further disseminated via earned and paid media in the form of newspapers, radio, and television commercials, high-profile awareness-based “street teams,” partner-based communications, social media, health fairs/expositions, and a variety of other avenues across the 7 county service areas for txt4health in Southeast Michigan. Although txt4health was available throughout the entire 7-county region in Southeast Michigan, particular dissemination emphasis was strategically focused on microtargeting 36 ZIP codes identified via third-party syndicated data as having high concentrations of individuals living with diabetes. At the outset, SEMBC staff members developed target enrollment estimates of at least 3000 individuals from the 7-county region.

In the Greater Cincinnati area, txt4health was launched with a kickoff event featuring Tim Ingram, Hamilton County Public Health Commissioner, and hosted by Kroger, a prominent grocery store chain in the region. Cincinnati efforts were coordinated by GCBC and Hamilton County Public Health and marketed with the slogan: “A text a day keeps the doctor away.” The Greater Cincinnati area txt4health pilot was disseminated via social media and health fairs/expositions, earned media, and outdoor and radio advertising, in addition to other outreach tactics across the 16-county GCBC catchment area. At the outset, GCBC staff members developed target enrollment estimates of at least 10,000 primary care patients with obesity, hypertension, and/or other prediabetic indicators. Additional information regarding the SEMBC and GCBC marketing strategies for txt4health have been more fully described previously [32].

### Program Evaluation Framework: RE-AIM

#### Overview

We utilized the RE-AIM framework to guide this evaluation, which is a model that provides a systematic approach to guiding the planning, evaluation, reporting, and review of health promotion interventions. Since its development in 1999 by Glasgow et al [35], the RE-AIM framework has been utilized extensively in the planning, evaluation, reporting, and review of health promotion interventions with a public health and community-based focus. The RE-AIM framework includes 5 dimensions: reach, efficacy, adoption, implementation, and maintenance. When considering each of these 5 dimensions independently, this systematic approach can lead to a more comprehensive understanding of the public health impact of a health promotion intervention. In this paper, we focus on the reach and adoption elements of RE-AIM.

#### Reach

Reach is an individual-level measure referring to the number of intended participants in a program [35]. By systematically considering participants who utilize a program in comparison to the target population, an understanding of the representativeness of a sample can be achieved. Within the present txt4health evaluation, reach refers to the individuals from subject service areas that initiated enrollment in txt4health.

#### Adoption

Adoption is an organizational measure that refers to the proportion and representativeness of settings that adopt a program [35]. Because txt4health targets individuals, not clinics or specific settings, we assessed adoption on an individual level. Within the present evaluation, adoption refers to individual participant enrollment, dropout, and engagement with txt4health.

### Procedures

We performed a retrospective data analysis of existing deidentified system-level usage data compiled through txt4health. The Wayne State University Institutional Review Board approved this study with a waiver of written consent for the retrospective records analysis.

### Measures

The system-level dataset contained information collected from participants including ZIP code, date of enrollment, and the date the participant dropped out (if applicable), as well as any available health profile (height and weight) and diabetes risk assessment (age, physical activity level, gender, gestational diabetes screen of having diabetes while pregnant or having given birth to a baby over 9 lb if participant was female, parental family history of diabetes, sibling history of diabetes, ethnicity, and smoking status) information. Because providing diabetes risk assessments to participants was the chief focus of txt4health, the number of participants who completed their risk assessment was our primary engagement measure of interest.

As secondary measures of engagement, we calculated the number of times a participant responded to their weekly weight and physical activity assessment, which was used to classify adherence with weight or exercise tracking. Participants who did not log any data were considered nonadherent to tracking, whereas participants who logged 4 times or less were classified as low adherers, 5 to 8 times were classified as medium adherers, and 9 or more times were classified as high adherers. In addition, from enrollment date to dropout date, we calculated the length of time that a participant was enrolled in txt4health. To better understand the influence of race, self-reported race data was recategorized into White and non-White classifications. Unfortunately, because Beacon staff members who were assisting potential participants with enrollment used the same website interface as an individual who was attempting to sign up via the website on their own, there was no way to determine which website enrollment initiations were assisted by Beacon staff and which were initiated by individual users.

### Data Analysis Strategies

We conducted descriptive statistics to describe participant enrollment, dropout, engagement, and participant characteristics based on health profile and diabetes risk assessment data. Continuous variables were expressed as mean (SD) and means were compared using 2-tailed unpaired independent samples *t* tests. Categorical data were displayed as frequencies and percentages, and chi-square tests were used for comparison. Multiple regression analyses were used to predict time spent in txt4health, whereas logistic regressions were used to predict program completion, weight goal setting, weekly activity tracking, and weekly weight tracking. Because of the highly skewed nature of weekly weight and activity tracking, we dichotomized these outcome variables into those participants who tracked 2 or more times versus those who did not. All regression models controlled for the continuous variables body mass index (BMI) and age, as well as the dichotomous variables gender, amount of exercise in a usual day, Beacon Community affiliation (Southeast Michigan or Greater Cincinnati), White or non-White race, and smoking status. Moreover, chi-square analyses were used to explore potential differences between participants affiliated with the Southeast Michigan or Greater Cincinnati Beacon Communities. Significance levels were set at a *P* value equal to or less than .05. All statistical analyses were carried out using STATA version 11.0 (StataCorp LP, College Station, TX, USA).

## Results

### Reach

**Figure 1:**
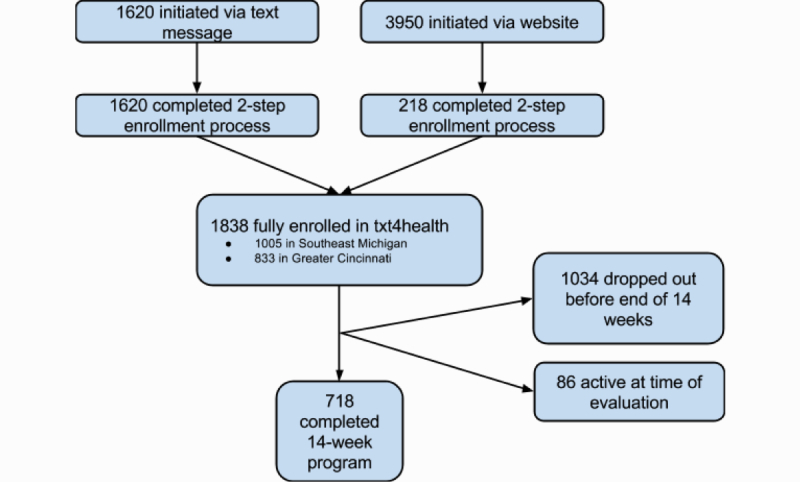
Participant flow through txt4health.

During the 10-month txt4health pilots, 5570 people initiated enrollment by using 1 of the 3 2-step enrollment processes: 1834 in Southeast Michigan and 3736 in Greater Cincinnati. In Southeast Michigan, enrollment was initiated equitably between text message (47.38%, 869/1834) and website sign-up (52.62%, 965/1834); whereas in Greater Cincinnati, 79.90% (2985/3736) of enrollments were initiated through the website. Across both pilots, 33.00% (1838/5570) of participants who initiated enrollment completed the 2-step enrollment process. Of participants who initiated enrollment via text message, 100.00% (1620/1620) completed the 2-step enrollment process, compared to 5.52% (218/3950) of participants who initiated enrollment via the website. Refer to Figure 1 for an illustration of participant flow.

In Southeast Michigan, 14.1% (136/965) of participants who initiated enrollment via website completed the 2-step enrollment process, compared to 2.75% (82/2985) in Greater Cincinnati. Website-initiated sign-ups included both participant-initiated website enrollments as well as participants who signed up with Beacon staff members, who later initiated enrollment on the website on behalf of the participant (which anecdotally comprise the vast majority of these enrollment initiations). Because both enrollment mechanisms used the same website interface, it is not possible to distinguish between participants who enrolled through either of these mechanisms.

In total, 71.00% (1305/1838) of participants furnished sufficient personal information to be categorized by diabetes risk level. From the data that was available, txt4health users were an average age of 41.2 years (SD 12.4), predominantly female (67.0%, 641/957), nonsmokers (83.36%, 912/1094) who engaged in little or no exercise on a typical day (62.48%, 701/1122), were obese with a BMI ≥30 kg/m^2^ (52.31%, 828/1583) and an average BMI of 32.1 kg/m^2^ (SD 9.2), and had an average weight of 203 lb (SD 60.0). The participant sample was racially diverse; 58.0% participants were White (472/814) and 35.4% were African American (288/814). In addition, of those with an assigned risk for developing diabetes based on the txt4health diabetes risk assessment, the majority were at high risk for developing diabetes (65.29%, 852/1305). See Table 2 for a complete breakdown of txt4health participant characteristics.

**Table 2:** Characteristics of txt4health users (N=1838).

Participant characteristic		Southeast Michigan	Greater Cincinnati	Total
**Gender, n**		509	448	957
	Female, n (%)	329 (64.6)	312 (69.6)	641 (67.0)
	Male, n (%)	180 (35.4)	136 (30.4)	316 (33.0)
**Age, n**		763	602	1365
	Mean (SD)^a^	42.9 (12.4)	39.1 (12.0)	41.2 (12.4)
**Ethnicity,** ^a^ **n**		421	393	814
	White, n (%)	193 (45.8)	279 (71.0)	472 (58.0)
	Black/African American, n (%)	197 (46.8)	91 (23.2)	288 (35.4)
	Hispanic/Latino, n (%)	13 (3.1)	8 (2.0)	21 (2.6)
	Asia/Pacific Islander, n (%)	10 (2.4)	6 (1.5)	16 (2.0)
	Other, n (%)	8 (1.9)	9 (2.3)	17 (2.1)
**Physically active, n**		601	521	1122
	Very active, n (%)	222 (36.9)	199 (38.2)	421 (37.5)
	Little or no exercise, n (%)	379 (63.1)	322 (61.8)	701 (62.5)
**Current weight category, n**		888	695	1583
	Underweight, n (%)	6 (0.7)	12 (1.7)	18 (1.1)
	Normal, n (%)	164 (18.5)	146 (21.0)	310 (19.6)
	Overweight, n (%)	238 (26.8)	189 (27.2)	427 (27.0)
	Obese, n (%)	480 (54.1)	348 (50.1)	828 (52.3)
**Parents family history,** ^b^ **n**		421	379	800
	No, n (%)	238 (56.5)	250 (66.0)	488 (61.0)
	Yes, n (%)	168 (39.9)	113 (29.8)	281 (35.1)
	Do not know, n (%)	15 (3.6)	16 (4.2)	31 (3.9)
**Sibling family history,** ^a^ **n**		421	379	800
	No, n (%)	311 (73.9)	318 (83.9)	629 (78.6)
	Yes, n (%)	86 (20.4)	39 (10.3)	125 (15.6)
	Do not know, n (%)	24 (5.7)	22 (5.8)	46 (5.8)
**Gestational diabetes screen,** ^c^ **n**		314	308	622
	No, n (%)	256 (81.5)	256 (83.1)	512 (82.3)
	Yes, n (%)	58 (18.5)	52 (16.9)	110 (17.7)
**Smoker,** ^a^ **n**		612	482	1094
	No, n (%)	531 (86.8)	381 (79.0)	912 (83.4)
	Yes, n (%)	81 (13.2)	101 (21.0)	182 (16.6)
**Risk category,** ^a^ **n**		725	580	1305
	High, n (%)	496 (68.4)	356 (61.4)	852 (65.3)
	Low, n (%)	229 (31.6)	224 (38.6)	453 (34.7)

^a^Significant difference (*P*<.001) found between Southeast Michigan Beacon Community (SMBC) and Greater Cincinnati Beacon Collaborative (GCBC) participants.

^b^Significant difference (*P*<.05) found between SMBC and GCBC participants.

^c^Gave birth to baby >9 lb.

###  Adoption

#### Overview

To assess txt4health adoption, we focused on 2 domains: participant dropout and engagement.

#### Participant Dropout/Retention

**Figure 2:**
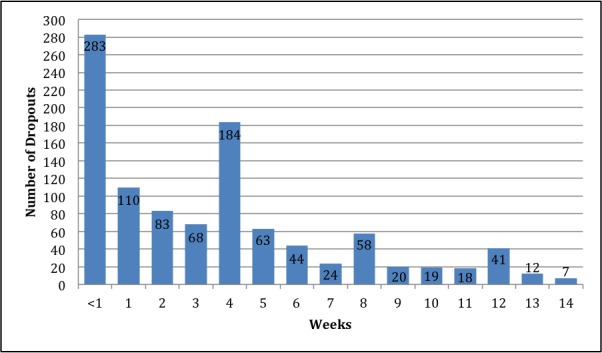
Participant dropout (n=1034) by week out of total number of participants (N=1838).

Of the 1838 participants who completed the 2-step enrollment process, 39.06% (718/1838) completed the program by receiving 14 weeks of messages, 56.26% (1034/1838) dropped out before the end of the 14-week program, and 4.68% (86/1838) were still active at the end of 2012 (Figure 1). Length of participation varied greatly, ranging from 0 to 48.7 weeks (median 8.6 weeks; mean 15.8 weeks, SD 15.8). In total, 718 participants were retained throughout the program. Participant dropout was highest within the first 7 days of the program. Of the 1034 people who dropped out, 27.37% (283/1034) exited the program before the participant completed the first week of txt4health. The bulk of participant dropout occurred within the first month of the program, with 70.41% (728/1034) occurring before the end of the fourth week. Although rates of participant dropout had a relatively steady decline over the course of the 14-week program, there were spikes in dropout rates during weeks 4, 8, and 12, which coincided with the scheduled messages reminding participants how to opt out of the program. See Figure 2 for a breakdown of participant dropout by week.

No significant differences were found in rates of dropout between participants who enrolled via text message versus other enrollment pathways. Using logistic regression and controlling for the continuous variables BMI and age, as well as the dichotomous variables gender, amount of exercise in a usual day, Beacon Community affiliation (Southeast Michigan or Greater Cincinnati), White or non-White race, and smoking status, race was the only participant characteristics that was a significant predictor of program completion, with non-White participants being more likely to complete the program than White participants (OR 2.35, 95% CI 1.66-3.31, *P*<.001). Regarding the length of time spent in txt4health, age (beta=.10, *P*=.048) and non-White race (beta=5.42, *P*<.001) were significant predictors of the number of weeks in the program with non-White participants completing more weeks of the program than White participants (mean 21.5 weeks, SD 14.7 and mean 16.0 weeks, SD 14.4, respectively; *t*_812_=5.28, *P*<.001).

#### Participant Engagement

To determine participation engagement, we looked at 4 different measures: diabetes risk assessment completion (primary measure of participant engagement), weight goal setting, weekly weight reporting, and weekly activity reporting (secondary measures of participant engagement). Of the 1838 participants who completed the 2-step enrollment process, 71.00% (1305/1838) completed the diabetes risk assessment. Moreover, 74.27% (1365/1838) set an initial weight loss goal at the beginning of the program. Using logistic regression analysis, several demographic variables were found to be significant predictors of weight goal setting. For each 1 kg/m^2^ increase in BMI, there was a 15% increase in likelihood of setting a weight goal (OR 1.15, 95% CI 1.09-1.21, *P*<.001), and for each 1 year increase in age, there was a 5% increase in likelihood of weight goal setting (OR 1.05, 95% CI 1.02-1.07, *P*<.001). In addition, females were more likely to set initial weight goals (OR 2.40, 95% CI 1.43-4.03, *P*=.001), as were White participants (OR 1.76, 95% CI 1.04-2.98, *P*=.04).

Over the course of the program, 89.17% (1639/1838) of enrolled participants tracked their current weight and 54.62% (1004/1838) tracked the number of days they had been physical active over the previous week at least once. When categorizing participants into low, medium, and high adherers to weekly weight and physical activity reporting, most participants were considered to be low adherers (logging 4 times or less over 14 weeks) for weight tracking (80.25%, 1475/1838), whereas most participants were either low adherers for activity tracking (30.58%, 562/1838) or not adherent at all, having never logged a weekly activity report throughout their program (45.38%, 834/1838). Although 13.60% (250/1838) of participants were highly adherent to weekly activity tracking, only 1.20% (22/1838) of participants were considered to be high adherers to weekly weight tracking.

When looking strictly at participants who completed the 14-week program (excluding dropouts and currently active participants), adherence rates for tracking weekly weights were low for program completers. Among txt4health users who received the full complement of tailored messages over the 14-week period, 71.4% (513/718) were categorized as low adherers for weight tracking and 6.8% (49/718) were completely nonadherent to weight tracking. Only 3.1% (22/718) of completers were highly adherent to weekly weight tracking. Patterns of tracking adherence varied for weekly activity reporting with 28.6% (205/718) of txt4health completers classified as low adherers and 22.7% (163/718) classified as completely nonadherent, yet 31.9% (229/718) were classified as high adherers, having logged a weekly activity amount at least 9 or more times. Using logistic regression, age was found to be a significant negative predictor of tracking weight 2 or more times (OR 0.98, 95% CI 0.97-1.00, *P*=.03) and physical activity 2 or more times (OR 0.98, 95% CI 0.96-0.99, *P*=001). In addition, White participants were more likely to track weekly weights 2 or more times than non-White participants (OR 1.6, 95% CI 1.15-2.26, *P*=.006). Finally, females were less likely than males to track weekly activity 2 or more times (OR 0.64, 95% CI 0.45-0.91, *P*=.01).

## Discussion

### Reach

The campaigns launching txt4health in Southeast Michigan and Greater Cincinnati were broadly disseminated through a variety of different mechanisms, including high-profile kickoff events and other launch-related activities that garnered significant local, regional, and even national media attention. Because of the tremendous amount of earned media press on top of other dissemination avenues used by the Beacon Communities, it is near impossible to estimate the number of people who were exposed to txt4health. Despite this, we can make a fair generalization that given the estimated population of Detroit and Cincinnati (approximately 700,000 and 300,000, respectively) [36,37], which represent only a small portion of the entire catchment areas of these Beacon Communities, the actual reach of txt4health, as estimated by the number of individuals who initiated the 2-step enrollment process (n=5570), was small. Undoubtedly, the number of people who enrolled in txt4health was a fraction of the number estimated by both Beacon Communities at the outset. Although we do not know why actual enrollment numbers were far lower than initial expectations, it is possible that either initial estimates were too high, or that campaign exposure and community response was lower than expected. Currently, the Crescent City Beacon Community in New Orleans, LA (the third Beacon Community to pilot txt4health), is attempting to shed light on this matter by undertaking efforts to measure campaign exposure of their own txt4health rollout within their community. Future work should seek to more acutely measure public health campaign exposure for txt4health by surveying a random sample of community members about txt4health awareness because the actual rates of program enrollment initiation may be a poor proxy for campaign reach. By obtaining better estimates of campaign exposure, it may be possible to tease out whether the campaigns promoting txt4health in the greater Detroit and Greater Cincinnati areas were not as effective as they could have been, or if consumer interest in txt4health was low. Despite the small enrollment numbers in comparison to the larger communities, according to the demographic information users supplied to the program for tailoring purposes, it appears that txt4health did reach the diverse target population at risk for developing type 2 diabetes.

Although the literature is sparse in regards to users of public health-focused text message programs, 2012 estimates from the Pew Internet & American Life Project reveal that only 9% of cell phone owners receive health/medical information via text message. In comparison to other cell phone owners, women and adults aged 30-64 years are more likely to receive health/medical text messages [38]. These trends were reflected in our findings. Although there exist examples in the literature of public health-focused mHealth interventions operating at scale, these often do not provide a good evidence base for what to expect in terms of user demographics of these types of interventions in the United States because they often originate internationally, such as the txt2stop program in the United Kingdom [24], or have only reported on regional usage of large programs, such as txt4baby [39]. Because the literature on broad-based public health-focused text messaging programs operating at scale is still underdeveloped, future research should strive to establish estimates of reach among the larger US population.

Distilling further down into the enrollment process reveals that only 33.00% of individuals who initiated enrollment completed both steps in the 2-step process. Previous work with Internet-mediated interventions has found similar patterns of potential participants not completing enrollment processes [40]. We attribute the fact that approximately two-thirds of our potential participants did not complete the enrollment process to the combination of the 2-step enrollment process and the website-initiated enrollment avenue. The 2-step enrollment process required participants to double opt-in to ensure that they were really willing to participate. Although slight, this process places additional burden on participants, which is exactly what this interaction was meant to do. Double opt-in procedures are common for text message programs because of regulatory requirements and are intended to ensure that participants really do want to participate in these programs.

Because of the added burden of the double opt-in enrollment process on participants, it seems logical that alleviating this burden by utilizing Beacon staff to initiate enrollment on behalf of potential participants, who signed up at community events, would yield higher rates of enrollment. After consideration of our results, we believe that this strategy was not effective; in fact, we believe this strategy artificially overinflated the number of individuals who did not complete the 2-step enrollment process. Although both the Southeast Michigan and Greater Cincinnati Beacon Communities used this strategy, this method was employed in Greater Cincinnati to a much greater extent, and the failure to shuttle potential participants through both steps of the 2-step enrollment process was particularly striking. As noted previously, approximately 80% of potential participants from Greater Cincinnati initiated enrollment via the website (the vast majority are attributed to participants signing up directly with Beacon staff who completed the online registration on behalf of the participant), yet only approximately 3% of these potential participants completed the 2-step enrollment process. Across both Beacon Communities, 100% of participants who initiated enrollment via text message completed the 2-step enrollment process, compared to 5.52% who initiated via website or Beacon staff member.

Although reasons for the failure to convert website-initiated potential participants into fully enrolled participants are not known, we believe that in many cases, potential participants who signed up with Beacon staff had low investment in program participation and had little or no intention to enroll. Whether it was because of peer pressure, etiquette, or other psychosocial reasons, we believe that the majority of those individuals were not likely to enroll, thus artificially lowering our enrollment completion rate. Moreover, there was no way for Beacon staff to verify that the phone numbers given by potential participants were legitimate, belonged to the participant themselves, or were for a cell phone and not a landline, all of which might have added to the failure to fully enroll some participants.

It is also likely that the failure of participants to fully enroll when initiating enrollment via website can be partially attributed to the lag time between the steps of the enrollment process. For those who enrolled via text message, the enrollment process was seamless and enrollment could be completed in under a minute although it contained multiple steps. For those who enrolled via the website, the enrollment process was broken up between a website or Beacon staffer sign-up, as well as a subsequent text message interaction. Individuals initiating enrollment themselves via the website were likely able to complete the 2-step enrollment process almost seamlessly by switching from one device (desktop computer, tablet, laptop, etc) to another (cell phone), and it is anticipated that very few of these people failed to complete the process. In contrast, those individuals who initiated enrollment through signing up with Beacon staff at health fairs likely experienced a considerable lag in the 2-step process where initial sign-up occurred in person, but the second text message confirmation step occurred at a later point in time. This lag in the enrollment process placed additional burden on participants and likely filtered out those with low investment by providing an opportunity to back out, or allowing potential participants to lose interest over time before enrollment. Enrollment via this mechanism may have been increased had mobile devices been used to initiate enrollment for potential participants in the moment, thereby generating a confirmation text message to the participant cell phone immediately.

Complex enrollment procedures have been previously related to barriers to enrollment [40,41]. For these reasons, we now believe that manually initiating enrollment on behalf of potential participants may boost the number of people who initiate enrollment, but this method is not likely to return a significant number of participants who fully enroll; in fact, it may overinflate the number of participants who do not complete enrollment. Because the majority of current literature focused on mHealth programs is still focused on relatively small demonstration projects versus larger public health-focused implementations, best practices for participant recruitment and enrollment are not known. Future work should seek to identify strategies to increase recruitment and enrollment in mHealth programs. In particular, future work should seek to determine the effectiveness of recruitment strategies originating from trusted sources and key opinion leaders, such as health care providers, health care organizations, other authority figures, all of which center prominently in models of persuasion and adoption, such as the 2-step flow of communication [42,43], the elaboration likelihood model [44], and the diffusion of innovations [45].

### Adoption

#### Participant Engagement

Nearly three-quarters of the enrolled participants completed the diabetes risk assessment, which was the primary focus of txt4health. It appears that txt4health was able to draw the intended target population, as 65.3% of participants with a risk profile were categorized as having high risk for developing diabetes. In terms of secondary measures, participant engagement in txt4health was varied, but this is expected given that many participants likely joined txt4health for the diabetes risk assessment and not the additional 14 weeks of tailored messages. Most participants set a weight loss goal (74.27%), and tracked their weight (89.17%) and physical activity (54.62%) at least once during the program. Adherence rates to weekly weight and physical activity tracking were variable. Although a greater proportion of participants tracked their weight at least once, as opposed to physical activity, there were a greater proportion of participants highly adherent to physical activity tracking (13.6%) than to weight tracking (1.20%). Among those who completed the 14 weeks of tailored messaging, these proportions increased to 31.9% highly adherent to physical activity tracking and 3.1% highly adherent to weight tracking. Reasons for these patterns of tracking are unknown, but it is possible that participants more frequently reported physical activity because they had engaged in some form of exercise over the previous week and had new information to report, whereas participants failed to engage in weekly weight tracking because they were not losing weight. Moreover, it is possible that some individuals may have no, or limited access, to a scale when requests to enter weekly weights were received. In the short term, physical activity goals that are related to the number of days of physical activity over the previous week are likely more easily achieved than weight loss goals. Future research should focus on determining strategies for increasing adherence to weekly tracking within mHealth programs because this has not yet been documented.

It should be noted that several changes were made to txt4health based on the lessons learned both during and after the 3 pilots, and the program has been significantly refined. To reduce barriers to enrollment, a need documented in this evaluation, several steps have been taken to streamline enrollment through partnerships with mobile carriers and health plans. To make txt4health relevant to a wider audience, the current iteration of txt4health now focuses on prevention more broadly because promotion and encouragement of health behaviors that are appropriate for decreasing risk for type 2 diabetes also apply to a broader audience. Finally, the program has taken steps to increase the level of participant engagement through mechanisms such as more interactive weight and exercise challenges, quizzes and other more interactive educational content, and encouraging and sending reminders for appropriate health screenings for diabetes and other conditions.

#### Dropout/Retention

Overall, txt4health retained 39.06% of participants throughout the 14-week program and lost 56.26% to drop out, which was most frequent within the first week of the program. Given that 71.00% of the enrollees completed the diabetes risk assessment, which was the primary purpose txt4health, it comes as no surprise that many people did not continue on to receive messages for 14 weeks. The bulk of participant dropout occurred before the end of the fourth week of the program, and followed a predictable pattern of declining dropout rates with each subsequent week of program participation, with small spikes in dropout rates in weeks 4, 8, and 12 corresponding to the weeks that opt-out instructions were automatically sent. Moreover, it is likely that dropout rates were underestimated. To formally drop out of txt4health, participants had to text “stop” to the program short code. Although slight, the act of formally dropping out of the program posed a burden on participants. It is likely that a subset of active users stopped reading messages during their program and never formally dropped out. This nonusage attrition is not directly measurable, but has been documented in the literature on Internet-mediated interventions [46-50]. Support for the presence of nonusage attrition may be found in the txt4health participants who never logged any weekly weight or physical activity data.

Although the literature documenting participant dropout in text message programs is sparse, the literature on Internet-mediated behavior change interventions documents lower retention rates, yet similar patterns of attrition [51-55]. Moreover, although recent estimates from 2012 reveal that smartphone users download an average of 41 apps to their smartphones [56], reports from the mobile analytics firm Localytics suggest that apps are often downloaded but abandoned after first use, with 22% of newly downloaded apps only being used once [57]. Although a text message-based intervention is not run via an app platform, attrition rates from that realm may provide some insight into what patterns of use may be expected from text message interventions. Given that we retained 39.06% of participants through at least 14 weeks, even allowing for some amount of nonusage attrition that is not accounted for, we believe that this program was comparable or better in regards to retention rates when compared to many Internet-mediated and app-based programs.

Although reasons for the high dropout rates exhibited in this investigation are unclear, we speculate several possible explanations. First, because the primary intention of txt4health was to provide participants with a diabetes risk assessment, it is possible that many dropout participants enrolled to determine their risk, but were not interested in receiving additional messages, prompting them to drop out shortly into the program. Another possible reason for attrition was the frequency and duration of messaging. Although 5-7 messages per week for 14 weeks does not sound like a high burden on participants, 7% of survey participants self-reported in the participant survey that there were too many messages sent within the program (see Part II of this evaluation). In addition, it is possible that too many messages may dilute the power of the messages that are sent. Little is currently known about the optimum frequency and duration of text message interventions, and although likely to be dependent on the nature of the program, future work should seek to better understand these factors.

Another potential reason for high dropout rates is the possibility that txt4health did not meet the expectations of all participants. For example, it is possible that a text message program is appropriate for conducting a one-time diabetes risk assessment for a broad base of participants, but that enthusiasm among a general audience for a 14-week behavior change intervention delivered via text message is tempered. In comparison to other successful, broad-based text message campaigns, such as txt4baby [58] targeting pregnant moms and txt2stop [24] targeting smoking cessation, that both have time-specific messages and information to convey, behavior changes regarding diet and physical activity are lifelong pursuits and individual needs regarding these behavior changes may not always be met with this sort of program. Also, participant needs may not have been met in regards to the subject matter. Although txt4health is focused on diabetes risk awareness and reduction, and was marketed as such by SEMBC with their Fighting D in the D campaign, this was not necessarily the case in the Greater Cincinnati area where GCBC’s marketing efforts billed “A text a day keeps the doctor away.” Without utilizing the word “diabetes” in the txt4health program name, or in the marketing slogan used by GCBC, it is possible that not all participants realized that txt4health was focused on diabetes.

Finally, it is also possible that dropout was spurred by costs associated with text messaging. Although text messaging is highly pervasive among American cell phone users, not all have access to free unlimited text messaging. This means that some individuals incur a nominal fee of upwards of US $0.20/message for each text message sent or received. In a program that targets at-risk individuals, many of which are low-income minorities, the delivery of a minimum of 5-7 text messages per week for 14 weeks could translate to significant participation costs. Although there exist examples of health-related text message programs that have worked with cell phone carriers to provide free text message delivery, such as text4baby [58], this was not the case for txt4health at the time of this evaluation.

### Strengths and Limitations

The major strength of this investigation is that we were focused on better understanding the use of a public health–focused text message program operating at scale. Through this work, we have started to identify the challenges with enrolling and maintaining participation of individuals in this type of program. This strength also happens to be a double-edged sword, in that it is precisely the nature of our community-based program that causes us to have a profound lack of information regarding our participants. Because we had to rely solely on participant self-reported data from diabetes risk assessments, we have no understanding of who failed to complete the 2-step enrollment process (because they never made it to that step) or why they failed to fully enroll. Furthermore, the self-reported demographic data gathered through the diabetes risk assessment was very basic and does not allow us to build a complete picture of the participants who used txt4health. Future work with large, public health–focused text message programs operating at scale should incorporate a stronger evaluation component from the outset so that more robust measures can be tracked from all stages of the program.

One of our goals was to measure the reach of txt4health. Because we had no way to measure the amount of campaign exposure within the community, and because we were not able to survey random community members about their awareness of the public health campaigns within their respective communities, it is impossible to determine an accurate measure of reach. Despite this, given the relatively small enrollment numbers in comparison to the population of the major metropolitan centers within the SEMBC and GCBC service area, it is safe to say that the reach of txt4health was small. Future work should seek to understand what type of enrollment rates and what patterns of participant engagement we could expect to see in similar text message programs marketed as a part of a larger public health campaign.

### Conclusions

This evaluation of the txt4health pilots in Southeast Michigan and Greater Cincinnati contributes greatly to the growing body of mHealth literature as it represents an effort to gain deeper understanding of individual use of a large-scale public health–focused text message–based intervention promoting behavior change. Although this type of program may not be appropriate for all, it is an appropriate delivery modality for reaching large populations, can retain a large proportion of users, and may provide some users with the tools needed to make necessary behavior changes.

## Abbreviations

BMI: body mass index
DPP: Diabetes Prevention Program
GCBC: Greater Cincinnati Beacon Collaborative
mHealth: mobile health
SEMBC: Southeast Michigan Beacon Community
